# Does Time Affect Patient Satisfaction and Health-Related Quality of Life After Reduction Mammoplasty?

**Published:** 2016-01-21

**Authors:** Wess A. Cohen, Peter Homel, Nima P. Patel

**Affiliations:** ^a^Department of Surgery, Maimonides Medical Center, Brooklyn, NY; ^b^Maimonides Medical Center, Brooklyn, NY; ^c^Division of Plastic Surgery, Department of Surgery, Maimonides Medical Center, Brooklyn, NY

**Keywords:** breast, breast reconstruction, BREAST-Q, patient-reported outcomes, quality of life

## Abstract

**Objective:** A total of 62,611 patients with breast hypertrophy underwent breast reduction surgery in 2013 in the United States to improve their symptoms and health-related quality of life. While multiple studies utilizing various outcome instruments demonstrate the efficacy of reductive surgery, it is presently unknown how the postoperative course affects patient satisfaction and health-related quality of life as measured by the BREAST-Q. Our objective was to determine the temporal relationship of patient satisfaction and health-related quality of life after reduction mammoplasty. **Methods:** Patients prospectively completed the BREAST-Q reduction mammoplasty module at 3 time points during their treatment: preoperatively, at less than 3 months postoperatively, and at more than 3 months (<12 months) postoperatively. A single surgeon (N.P.P.) performed all of the breast reduction procedures. **Results:** Each time point contained 20 questionnaires. Mean preoperative BREAST-Q scores were significantly lower than scores at the less than 3-month postoperative time point for the scales Satisfaction With Breasts, Psychosocial Well-being, Sexual Well-being, and Physical Well-being (*P* < .001). There was no significant difference in BREAST-Q scores between the postoperative time points in these measures. **Conclusion:** Breast reduction surgery offers a vast improvement in patients’ satisfaction and health-related quality of life that is maintained throughout the postoperative period. These findings can assist surgeons in managing patient expectations after reduction mammoplasty and help improve the probability of obtaining prior authorization for insurance coverage.

Breast reduction surgery is often undertaken because of gross dissatisfaction with the appearance of one's breasts, psychosocial embarrassment, or physical discomfort. In 2013, a total of 62,611 women underwent reduction mammoplasty in the United States.^1^ While various outcomes instruments have been used to demonstrate the efficacy of reductive surgery in alleviating symptoms,^2^^-^5 only 4 studies have utilized the BREAST-Q,^6^^-^9 the current standard for procedure-specific patient-reported outcomes (PROs) in breast surgery.

Patient satisfaction and health-related quality of life (HR-QOL) are high at various postoperative time points after reduction mammoplasty.^6^^,^^8^ In the only prospective study utilizing the BREAST-Q for reduction mammoplasty, Coriddi et al^7^ found that breast reduction surgery significantly improved patients’ satisfaction with the appearance of their breasts, as well as their psychosocial, physical, and sexual well-being, as early as 6 weeks postoperatively compared with preoperative levels. Other studies have demonstrated high levels of patient satisfaction and HR-QOL but were not prospective and did not investigate how time from surgery impacts PROs.^6^^,^^8^

Utilizing a prospective cohort, our objective was to investigate whether time from reduction mammoplasty impacts patient satisfaction and HR-QOL. We hypothesize that patient satisfaction and HR-QOL will change during the postoperative period. With this information, surgeons and their staff will be better able to assist patients in managing their postoperative expectations.

## METHODS

After approval by the institutional review board at Maimonides Medical Center in Brooklyn, NY, all patients seen by a single surgeon (N.P.P.) between June 2012 and January 2014 were asked to prospectively complete the BREAST-Q reduction/mammoplasty questionnaire preoperatively and again at each postoperative follow-up visit.

The BREAST-Q is a validated breast surgery–specific PRO instrument.^10^ Raw scores were converted to a 0 to 100 scale by the Q-score program. Larger numbers equate to a stronger agreement with the question posed and translate to increased satisfaction or HR-QOL. The BREAST-Q consists of 4 scales, which may be completed both before and after breast surgery: Satisfaction with Breasts; Psychosocial Well-being; Sexual Well-being; and Physical Well-being. In addition, there are 5 scales, which can be administered only after surgery: Satisfaction With Outcome; Nipples; Information; Plastic Surgeon; Medical Team; and Office Staff.

### Statistical analysis

Data were described in terms of the mean for normally distributed variables and as frequency (percent) for categorical variables. Mixed-model linear regression was used to compare BREAST-Q scores preoperatively, immediate postsurgery (≤3 months after surgery), and extended postsurgery (>3 months after surgery). It allows for inclusion of all respondents regardless of missing data based on the assumption of data missing at random. A mean contrast test made specific comparison between preoperative and immediate postsurgery and immediate postsurgery versus extended postsurgery. A value of *P* < .05 was considered significant.

## RESULTS

Fifty-one patients underwent breast reduction surgery from June 2012 to January 2014. Of those, 27 patients completed the BREAST-Q at a minimum of 1 time point. Of those 27, 20 patients completed questionnaire at each time point: a 74% response rate. Patient demographics are shown in Table 1.

**Figure 1:**
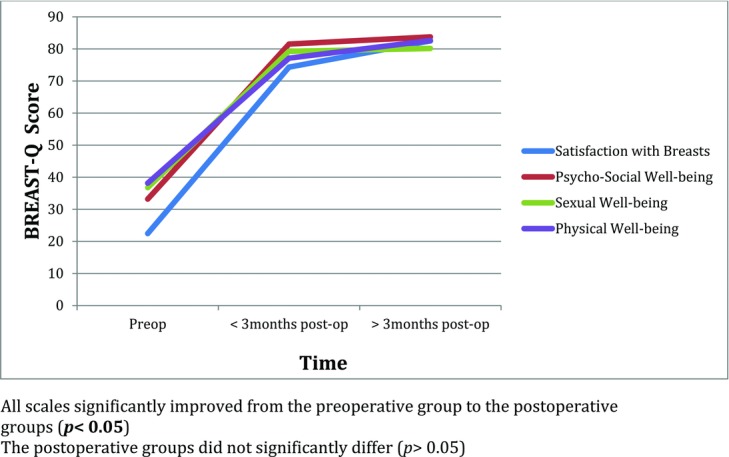
Mean change in BREAST-Q score over time.

Mean BREAST-Q scores significantly improved from preoperative levels to less than 3 months postoperatively in mean satisfaction with breasts, 22 versus 74 (*P* < .001); mean psychosocial well-being, 33 versus 82 (*P* < .001); mean sexual well-being, 37 versus 79 (*P* < .001); and mean physical well-being, 38 versus 77 (*P* < .001). There was no significant difference in BREAST-Q scores between the postoperative time points in these measures (Fig 1).

Table 2 depicts BREAST-Q scores in the postoperative period alone. Time from surgery did not significantly affect satisfaction with outcome or satisfaction with nipples (Table 2).

## DISCUSSION

The Institute of Medicine's 2001 report, *Crossing the Quality Chasm*, highlighted the importance of patient information in health care.^11^ While clinical experience can assist physicians in anticipating clinical outcomes and changes over time, PROs directly measure patients’ satisfaction and HR-QOL. We chose to use the BREAST-Q reduction mammaplasty module because it is comprehensive and procedure-specific. We followed patients for up to 1 year postoperatively to provide surgeons with the likely evolution of satisfaction and HR-QOL during the period of most patient interaction.

In the context of shared decision making, patients seek meaningful information about how they will feel postoperatively. Breast reduction surgery is known to improve QOL and reduce pain within a few months of surgery.^12^^-^16 Furthermore, these benefits as well as aesthetic satisfaction are present many years after surgery.^8^^,^^15^^,^^17^^-^21 However, no studies have used the BREAST-Q prospectively to illustrate the value of breast reduction surgery and the evolution of PROs.

Patient satisfaction and HR-QOL sharply improve after surgery (Fig 1). Our immediate postoperative data support those of Coriddi^7^ et al, who showed a marked improvement from baseline in satisfaction and HR-QOL at 6 weeks after surgery.^7^ At this early stage, while patients experience the physical relief of lost breast weight, they are still in the midst of the healing process. Therefore, these patients may not fully realize how the surgery impacts different aspects of their lives.

The significant increase in HR-QOL and patient satisfaction after surgery is maintained for at least 1 year (Fig 1). Prior studies have shown that patient satisfaction and HR-QOL improve at 12 months after surgery compared with preoperative levels using alternative PRO instruments.^15^^,^^22^ In addition, patients may continue to benefit from reduction mammoplasty many years postsurgery.^8^^,^^23^ While Thoma et al^24^ presented similar results and demonstrated that the main benefit of reduction mammoplasty is realized within 1 month after surgery, it is possible that the PRO instrument used was not sensitive to patient and clinical changes. Our study is the first to prospectively follow patients at multiple time points in the postoperative period utilizing the BREAST-Q.

By continually demonstrating a sustained relief of physical symptoms and improvement of HR-QOL, plastic surgeons can assist in promoting broader insurance coverage for reduction mammoplasties. Insurance companies are often inconsistent in their coverage of breast reductions.^25^ Coverage stipulations range from a minimum age to a minimum preoperative breast size to a minimum resection weight. Similar ambiguities that resulted in decreased coverage were present for breast reconstruction until the Women's Health and Cancer Rights Act of 1998 mandated all payer coverage for postmastectomy reconstruction.^26^ Our study shows significant improvement of physical well-being that is maintained in the postoperative period after reduction mammoplasty.

While this study has the advantage of being prospective and using the BREAST-Q, it does suffer from some limitations. Not all patients completed the BREAST-Q at each time point. It is conceivable that either the most or least satisfied patients completed the questionnaire at different points. This can be addressed by more diligent BREAST-Q administration by the surgeon and office staff. In addition, there is a lack of standardized and long-term follow-up. In the future, addressing these limitations, as well as providing a large multicenter patient population, will afford greater power and generalizability of the results.

## CONCLUSION

Breast reduction surgery offers a vast improvement in patients’ satisfaction and HR-QOL that is maintained throughout the postoperative period. These findings can assist surgeons in managing patient expectations after reduction mammoplasty and help improve the probability of obtaining prior authorization for insurance coverage.

## Figures and Tables

**Table 1:** Patient Characteristics (n = 27)

Characteristic	Value
Age, y	43.52*
BMI, kg/m^2^^†^	31.58*
Height, in	63.90*
Weight, lb	183.14*
Tissue Resection	
Right tissue removed, g	921.00*
Left weight tissue, g	924.96*
Technique	
Wise pattern	23 (85.19)^‡^
No vertical scar	4 (14.81)^‡^
Complications	
Partial tissue necrosis	1 (3.70)^‡^
Wound dehiscence	3 (11.11)^‡^

*Mean.

^†^BMI, body mass index.

^‡^Frequency.

**Table 2:** Postoperative mean BREAST-Q satisfaction scores

	≤3 mo postoperatively	>3 mo postoperatively	*P*
Outcome	82.00	89.69	.20
Nipples	81.53	89.77	.29
